# Bovine tuberculosis in the Middle East and North Africa: a systematic review and meta-analysis on prevalence and *Mycobacterium bovis* clonal complexes

**DOI:** 10.3389/fvets.2026.1861602

**Published:** 2026-06-22

**Authors:** Tsegaye Shamebo, Aboma Zewude, Temesgen Mohammed, Nabeeha Abdelgaleel Hassan, Mohammed Saleh Albreiki, Yassir Mohammed Eltahir, Atef Oreiby, Robert Barigye, Markos Tibbo, Ali Artaman, Gobena Ameni

**Affiliations:** 1 Department of Health Sciences, Natural and Health Sciences, Zayed University, Academic City, Dubai, United Arab Emirates; 2 Department of Veterinary Medicine, College of Agriculture and Veterinary Medicine, United Arab Emirates University, Al Ain, United Arab Emirates; 3 Environmental and Occupational Health, Institute of Public Health, College of Medicine and Health Sciences, United Arab Emirates University, Al Ain, United Arab Emirates; 4 Biosecurity Affairs Division, Abu Dhabi Agriculture & Food Safety Authority, Mohamed Bin Zayed City, Abu Dhabi, United Arab Emirates; 5 Animals Extension and Health Services Division, Abu Dhabi Agriculture and Food Safety Authority (ADAFSA), Abu Dhabi, United Arab Emirates; 6 Department of Animal Medicine, Faculty of Veterinary Medicine, Kafrelsheikh University, Kafr El-Sheik, Egypt; 7 Subregional Office for the Gulf-Cooperation Council States and Yemen, Food and Agriculture Organization of the United Nations, Abu Dhabi, United Arab Emirates

**Keywords:** bovine tuberculosis, *M. bovis* clonal complex, MENA, meta-analysis, pooled prevalence

## Abstract

**Introduction:**

Bovine tuberculosis (bTB) is endemic in the Middle East and North Africa (MENA) region causing major threat to dairy production and milk consumers. This paper presents a comprehensive systematic review and meta-analysis of bTB prevalence and *Mycobacterium bovis* clonal complexes in the MENA region based on studies published between 2000 and 2025.

**Methods:**

A comprehensive literature search was conducted in PubMed, Scopus, Web of Science, Embase, and relevant regional databases. Thirty-eight studies met the criteria and included in the meta-analysis. Random-effects meta-analysis was used to estimate pooled prevalence. Additional analyses were done on sensitivity, meta-regression, and publication bias.

**Results:**

A total of 38 studies comprising 100,094 animals and 2,830 confirmed bTB cases were included in the meta-analysis. Screening test-based pooled prevalence was 5.3% (95% CI: 2.5–10.7%) while confirmatory test-based pooled prevalence was 2.3% (95% CI: 1.4–3.7%). Based on confirmatory test, higher pooled prevalence was recorded in the Middle East (5%) than in the North Africa (2%) while the pooled prevalence in Egypt (1%) was lower than the pooled prevalence (5%) of the other MENA countries. High heterogeneity (*I*^2^ > 99%) was observed among prevalence reported by the confirmatory test and the sources of heterogeneity were identified to be country’s prevalence report and sample size based on meta-regression analysis. Confirmatory test-based sensitivity analysis demonstrated robustness the pooled prevalence while Egger’s regression test showed the presence of publication bias (*p* = 0.001) that was confirmed by Trim and Fill analysis which suggested the presence of missing studies that lead to underestimation of pooled prevalence. Molecular analysis of 590 *M. bovis* isolates showed that the European 2 (Eu2) clonal complex was predominant (63.6%), followed by Eu1 (9.7%) and Eu3 (6.6%), while 20.2% of isolates could not be assigned to known clonal complexes. The most reported spoligotypes were SB0120, SB0121, and SB0134.

**Conclusion:**

Low pooled prevalence with high heterogeneity was recorded in the MENA region. The dominance of European clonal complexes of *M. bovis* could suggest historical its introduction with cattle importation. Substantial gaps in prevalence warrants for more surveillance studies in order to establish the national prevalence in each country.

**Systematic review registration:**

Epidemiology and Genetic Diversity of Mycobacterium bovis in Animals in the Middle East and North Africa: A Systematic Review and Meta-Analysis [CRD420261326357].

## 1 Introduction

Bovine tuberculosis (bTB) is a chronic infectious disease of cattle primarily caused by *Mycobacterium bovis* (*M. bovis*), a member of the *Mycobacterium tuberculosis* complex (MTBC). This pathogen can infect a broad range of hosts, including livestock, wildlife, and humans, making it an important concern within the One Health framework (1, 2). In cattle, transmission mainly occurs through inhalation of infectious aerosols, whereas human infection is commonly associated with the consumption of contaminated unpasteurized dairy products or direct occupational exposure to infected animals (3, 4).

Globally, bTB remains one of the most important zoonotic diseases affecting animal health, public health, and the productivity of animal production. The disease is widely distributed across Africa, Asia, Latin America, and parts of the Middle East, where surveillance and control programs are often limited (5, 6). It has been estimated that zoonotic tuberculosis caused by *M. bovis* accounts for thousands of human tuberculosis (TB) cases annually, particularly in low- and middle-income countries where close contact between humans and livestock is common and milk pasteurization is inconsistently practiced (3, 7). Besides, bTB causes substantial economic losses through reduced productivity, trade restrictions, carcass condemnation, and costs associated with testing and eradication programs (5, 8).

Although several high-income countries have significantly reduced disease prevalence through test-and-slaughter strategies and strict surveillance systems, eradication remains difficult in many endemic settings. The persistence of infection is influenced by factors such as wildlife reservoirs, unrestricted animal movement, inadequate veterinary infrastructure, and inconsistent disease control measures (5, 9). Furthermore, zoonotic tuberculosis caused by *M. bovis* is frequently underdiagnosed because routine laboratory methods often fail to distinguish it from infections caused by *Mycobacterium tuberculosis* (*M. tuberculosis*) (3, 10).

The epidemiological context of the Middle East and North Africa (MENA) region is particularly favorable for the persistence and spread of bTB. Livestock production systems vary widely across the region, ranging from intensive dairy farming to extensive pastoral systems, frequently accompanied by transboundary livestock movement. Such movements contribute substantially to the dissemination of *M. bovis* strains across national borders (11, 12). Moreover, inconsistencies in surveillance systems, testing strategies, culling practices, and animal movement regulations continue to limit effective disease control. Consequently, available epidemiological data from MENA countries remain fragmented and inconsistent, making accurate regional assessment difficult (12).

Accurate estimation of bTB prevalence is essential for designing effective control programs; however, prevalence estimates vary considerably among studies because of differences in sampling approaches, animal populations, and diagnostic methods (10). Common diagnostic techniques include the tuberculin skin test (TST), interferon-gamma release assays (IGRAs), postmortem meat inspection, bacteriological culture, and molecular assays such as polymerase chain reaction (PCR). Differences in the sensitivity and specificity of these methods complicate direct comparison between studies and emphasize the need for systematic evidence synthesis (4, 13).

Recent advances in molecular epidemiology have improved understanding of the transmission dynamics and evolutionary relationships of *M. bovis*. Genotyping approaches based on genomic deletions and single nucleotide polymorphisms (SNP) have enabled the classification of *M. bovis* into several clonal complexes, including European 1 (Eu1), European 2 (Eu2), African 1 (Af1), and African 2 (Af2). These lineages demonstrate distinct geographic distributions and are strongly associated with historical cattle trade and livestock movement patterns (14, 15). Emerging genomic evidence also indicates that multiple clonal complexes may circulate simultaneously within the same region, reflecting complex transmission networks involving livestock, wildlife, and humans.

Although studies conducted in different MENA countries have reported variable prevalence estimates and diverse *M. bovis* strains (16), major differences in study design, sampling strategies, and diagnostic practices remain. As a result, a comprehensive regional understanding of the epidemiology and molecular diversity of bTB is still lacking.

Therefore, the objective of this study was to systematically review and quantitatively synthesize available evidence on the prevalence of bTB in the MENA and to characterize the distribution of *M. bovis* clonal complexes reported in the region. By integrating epidemiological and molecular findings, this study aims to support evidence-based and regionally coordinated strategies for the control of this transboundary zoonotic disease.

## 2 Methods

### 2.1 Study design

This review included cross-sectional studies that estimated the prevalence of bTB in the MENA region. These studies were selected as they provide estimates of bTB occurrence at a specific point in time, which is consistent with the objective of this meta-analysis to synthesize and assess the available epidemiological evidence on bTB.

### 2.2 Participants

This review was conducted on studies that were done mainly in cattle. In addition, some studies that were conducted on mixed species (cattle and buffalo) were also included in the review for assessing the prevalence of bTB in these animals in countries of the MENA region.

### 2.3 Systematic review protocol

This study was conducted as a systematic review and meta-analysis following the PRISMA 2020 reporting guidelines (17). Details of the search strings and the systematic review and meta-analysis protocol have been published in PROSPERO (CRD420261326357) and can be accessed using its reference number.

### 2.4 Inclusion and exclusion criteria

Studies that were conducted in countries within the MENA region and published between 2000 and 2025 were included. The included studies were observational studies conducted in cattle or cattle and buffalo mixed species. Studies that reported prevalence on the basis of confirmatory were primarily considered as most the studies conducted in the MENA region used confirmatory test uniformly while they used several different types of screening tests. The minimum sample size of included studies was 100 or greater than 100. Studies that did not fulfil the above criteria were not included.

### 2.5 Data sources

A comprehensive literature search was performed in major electronic databases, including PubMed/MEDLINE, Scopus, Web of Science, and Embase, to identify studies published between 1 January 2000 and 31 December 2025. To improve regional coverage, additional sources such as African Journals Online and Iran Medex were also consulted when available. Reference lists of all eligible articles and relevant reviews were further screened to identify additional studies not captured during the initial search.

The search strategy used combinations of relevant keywords and Boolean operators related to bovine tuberculosis. Key terms included “*Mycobacterium bovis*,” “bovine tuberculosis,” and “animal tuberculosis,” combined with epidemiological terms such as “prevalence,” “epidemiology,” and “distribution.” These were linked with geographic descriptors including “Middle East,” “North Africa,” “MENA,” and specific country names (e.g., Egypt, Iran, Morocco, Sudan, Algeria, Tunisia, Turkey, and Saudi Arabia). Studies conducted between 2000 and 2025 involving cattle or buffalo with a sample size greater than 100 were considered eligible for inclusion. Search syntax was adjusted as required for each database.

### 2.6 Study selection and data extraction

Data were extracted using a standardized Microsoft Excel template that captured the author and publication year, country, animal species, study design, sample size, number of *Mycobacterium bovis*–positive cases, diagnostic methods, study period, molecular characterization, and reported risk factors. Where studies reported multiple diagnostic outcomes, only prevalence estimates based on culture and/or PCR confirmation were extracted for meta-analysis.

All retrieved records were imported into a reference management software, and duplicates were removed. Two reviewers independently screened titles and abstracts, followed by full-text assessment based on predefined inclusion and exclusion criteria. Discrepancies were resolved through discussion or consultation with a third reviewer. The study selection process was summarized using a PRISMA flow diagram.

Methodological quality and risk of bias were assessed using the Joanna Briggs Institute (JBI) Critical Appraisal Checklist for Prevalence Studies. Only studies scoring above 7 points, indicating low risk of bias, were included. Sensitivity analyses were performed to evaluate the influence of higher-risk studies on pooled estimates.

### 2.7 Research indicator

The primary outcomes of interest of the study were prevalence of bTB and distribution of *M. bovis* clonal complexes in the MENA region.

### 2.8 Data analysis

Eligible studies were first described narratively to present the prevalence, distribution, and molecular features of *M. bovis* in the MENA region. Radom model meta-analysis was performed using R (version 4.5.3) with the meta and metafor packages to estimate the pooled prevalence of bTB. Heterogeneity among studies was assessed using Cochran’s Q test and the I^2^ statistic. Subgroup analyses were conducted only when sufficient numbers of studies were available within each category. The analyses primarily focused on diagnostic methods, countries and host species to investigate potential sources of heterogeneity. Due to the limited number of eligible studies from some regions and categories, certain subgroup findings were interpreted with caution. Publication bias was evaluated using funnel plot asymmetry and Egger’s regression test. Sensitivity analyses were performed to evaluate the robustness of the findings.

## 3 Results

### 3.1 Selected studies and their characteristics

**Figure 1:**
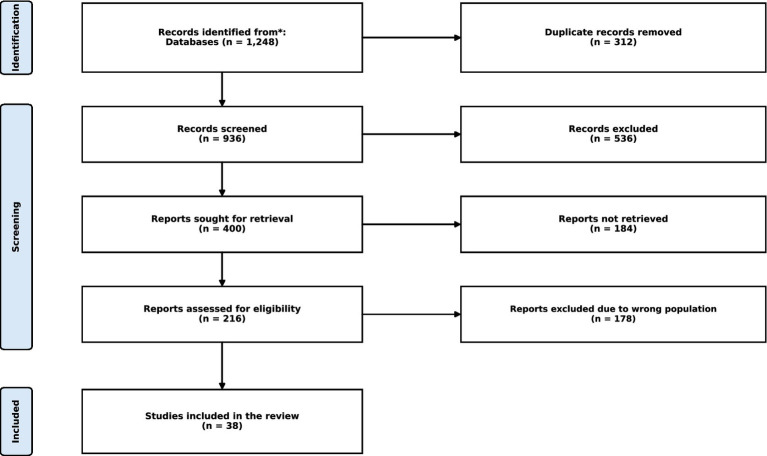
PRISMA flow diagram of study selection for the systematic review and meta-analysis of bTB in animals across the MENA region. The figure shows the PRISMA-based study selection process. From 1,248 identified records, 312 duplicates were removed and 936 records were screened. After exclusions and eligibility assessment, 38 studies were finally included in the systematic review and meta-analysis.

Out of 1,248 records identified through database and manual searches, 50 studies met the inclusion criteria for systematic review. Out of the 50 selected studies, 38 studies provided sufficient data for meta-analysis (Figure 1). The PRISMA flow diagram illustrates the screening steps of eligible studies. In addition, the PRISMA checklist is also attached to show the its consideration in the manuscript preparation (Supplementary material S1). Only eight of the 21 MENA countries contributed studies to this review. Egypt contributed 46% (23/50), Algeria (14%), Iraq (10%), Iran (8%), Morocco, Turkey and Tunisia (6%), and Sudan (4%) (Table 1).

**Table 1:** Characteristics of the studies conducted on bTB in the Middle East and North Africa between 2000 and 2025.

**S/N**	**Author**	**Year**	**Country**	**Study design**	**Species**	**Sample size**	**Screening test**	**Case**	**Types of sample tested**	**Confirmatory test**	**Case**	**Genotyping method**
1	Abdelaal et al. (29)	2019	Egypt	Observational study	Cattle	7,064	SICST	242	Tissue	Culture + PCR	31	MIRU-VNTR; WGS
2	Abdelsadek et al. (30)	2020	Egypt	Observational study	Cattle	1,464			Milk, Lymph nodes	Culture + PCR	127	
3	Al-Fattli (31)	2016	Iraq	Observational study	Cattle	119	TST	42	Blood and milk	NA	42	
4	Algammal et al. (32)	2019	Egypt	Observational study	Cattle	2,600	SIDT	40	Tissue	Culture+ PCR	40	
5	Al-Thwani and Al-Mashhadani (33)	2016	Iraq	Observational study	Cattle	300			Tissue	Culture+ PCR	4	
6	Alwathnani et al. (34)	2012	Egypt	Observational study	Cattle +Buffalo	6,205	TST	190	Tissue, Milk, Blood	Culture+ PCR	16	
7	Asil et al. (35)	2013	Sudan	Observational study	Cattle	6,680			Tissue	Culture+ PCR	12	
8	Aydın et al. (36)	2012	Turkey	Observational study	Cattle	145			Milk	Culture + PCR	1	
9	Barak (37)	2012	Iraq	Observational study	Cattle	850	CITT	373	Serum, Milk, swab nasal, tissue samples from cattle	Culture	100	
10	Belakehal et al. (38)	2022	Algeria	Observational study	Cattle	3,848			Tissue	Culture +PCR	59	Spoligotypin; MIRU-VNTR
11	Ben Kahla et al. (39)	2011	Tunisia	Observational study	Cattle	102	SCITT	102	Milk	Culture	5	IS6110-RFLP; MIRU-VNTR; Spoligotyping
12	Borham et al. (40)	2022	Egypt	Observational study	Cattle +Buffalo	750			Tissue	Culture+ PCR	9	
13	Damene et al. (41)	2020	Algeria	Observational study	Cattle	3,546			Tissue	Culture+ PCR	174	Spoligotyping
14	Elnaker et al. (42)	2019	Egypt	Observational study	Cattle	10,903			Tissue	Culture+ PCR	1,112	
15	Elsayed (43)	2019	Egypt	Observational study	Cattle +Buffalo	2,100	STST	65	Tissue	Culture+ PCR	61	
16	Elsayed and Amer (44)	2019	Egypt	Observational study	Cattle +Buffalo	3,700			Tissue	PCR	54	MIRU-VNTR
17	Elsayed et al. (45)	2022	Egypt	Observational study	Cattle +Buffalo	6,000			Tissue	Culture+ PCR	23	
18	Elsohaby et al. (46)	2020	Egypt	Observational study	Cattle	2,710	SCT	44	Milk	Culture+ PCR	56	
19	Hassan et al. (47)	2018	Egypt	Observational study	Cattle	2,650	CTST	63	Tissue	Culture	47	
20	Karamian et al. (48)	2022	Iran	Observational study	Cattle	1700	CITT	144	Tissue	PCR	44	
21	Lamine-Khemiri et al. (49)	2014	Tunisia	Observational study	Cattle	100			Tissue	Culture+ PCR	27	Spoligotypin; MIRU-VNTR
22	Lobna et al. (50)	2015	Egypt	Observational study	Cattle	420	SITT	8	Milk	Culture+ PCR	1	
23	Manal and Gobran (51)	2008	Egypt	Observational study	Cattle	704			Tissue	Culture+ PCR	5	
24	Mohamed et al. (52)	2009	Egypt	Observational study	Cattle	745			Tissue	Culture	12	
25	Mosavari et al. (53)	2011	Iran	Observational study	Cattle	213			Tissue	Culture + PCR	56	
26	Mossad et al. (54)	2009	Egypt	Observational study	Cattle	3,000	SITT	36	Tissue	PCR	90	
27	Moussa et al. (55)	2011	Egypt	Observational study	Cattle	1,180			Tissue	Culture+ PCR	20	
28	Ramadan et al. (56)	2012	Egypt	Observational study	Cattle	3,347			Tissue	Culture+ PCR	21	
29	Sabry and Elkerdasy (57)	2014	Egypt	Observational study	Cattle	300	TST	35	Blood	Culture + PCR	13	
30	Sahraoui et al. (58)	2009	Algeria	Observational study	Cattle	7,250			Tissue	Culture	88	Spoligotyping; MIRU-VNTR
31	Shereen et al. (59)	2015	Egypt	Observational study	Cattle	2,935			Tissue	Culture	39	
32	Solmaz et al. (60)	2009	Turkey	Observational study	Cattle	210	TST	3	Nasal, Milk	PCR	3	
33	Sulieman and Hamid (61)	2002	Sudan	Observational study	Cattle	120			Lymph nodes, Tissue	Culture+ PCR	25	IS6110-RFLP
34	Tadayon et al. (62)	2008	Iran	Observational study	Cattle +Buffalo	628			Tissue	Culture + PCR	199	IS6110-RFLP; MIRU-VNTR
35	Tazerart et al. (63)	2021	Algeria	Observational study	Cattle	928			Tissue	Culture + PCR	13	WGS
36	Tuzcu and Köksal (64)	2020	Turkey	Observational study	Cattle	5,018			Tissue	Culture	32	Spoligotyping; MIRU-VNTR
37	Yahyaoui-Azami et al. (65)	2017	Morocco	Observational study	Cattle	8,658			Tissue	Culture+ PCR	144	
38	Zahran et al. (66)	2014	Egypt	Observational study	Cattle +Buffalo	902	STST	36	Tissue	Culture+ PCR	25	IS6110-RFLP

The highest prevalence was reported from Iran (31.7%), followed by Tunisia (average 15.8%) and Iraq (average 11.4%), which indicate high burden of bTB in these countries. On the other hand, low prevalence values were reported from Morocco (average 2.4%), Algeria (average 2.14%), and Saudi Arabia (average 2.4%). Moreover, the least prevalence values were reported from Turkey (average 0.7%), Sudan (average 0.5%) and Egypt (average 0.05%). While in contrast, no eligible studies were identified from the remaining countries of the MENA region.

Most studies were conducted in cattle while a few were performed on mixed species (cattle and buffalo). Although the diagnostic tests used included tuberculin skin test (TST), ELISA, culture, PCR, and genotyping methods such as spoligotyping, MIRU-VNTR, and whole-genome sequencing (WGS), meta-analysis mainly was based on prevalence reported using culture and or PCR, as most of the studies used these methods culture and or PCR. In addition, pooled prevalence was also estimated based on TST for 15 of the 38 studies that used TST for screening of bTB.

### 3.2 . Pooled prevalence of bovine tuberculosis on the basis of screening test

**Figure 2:**
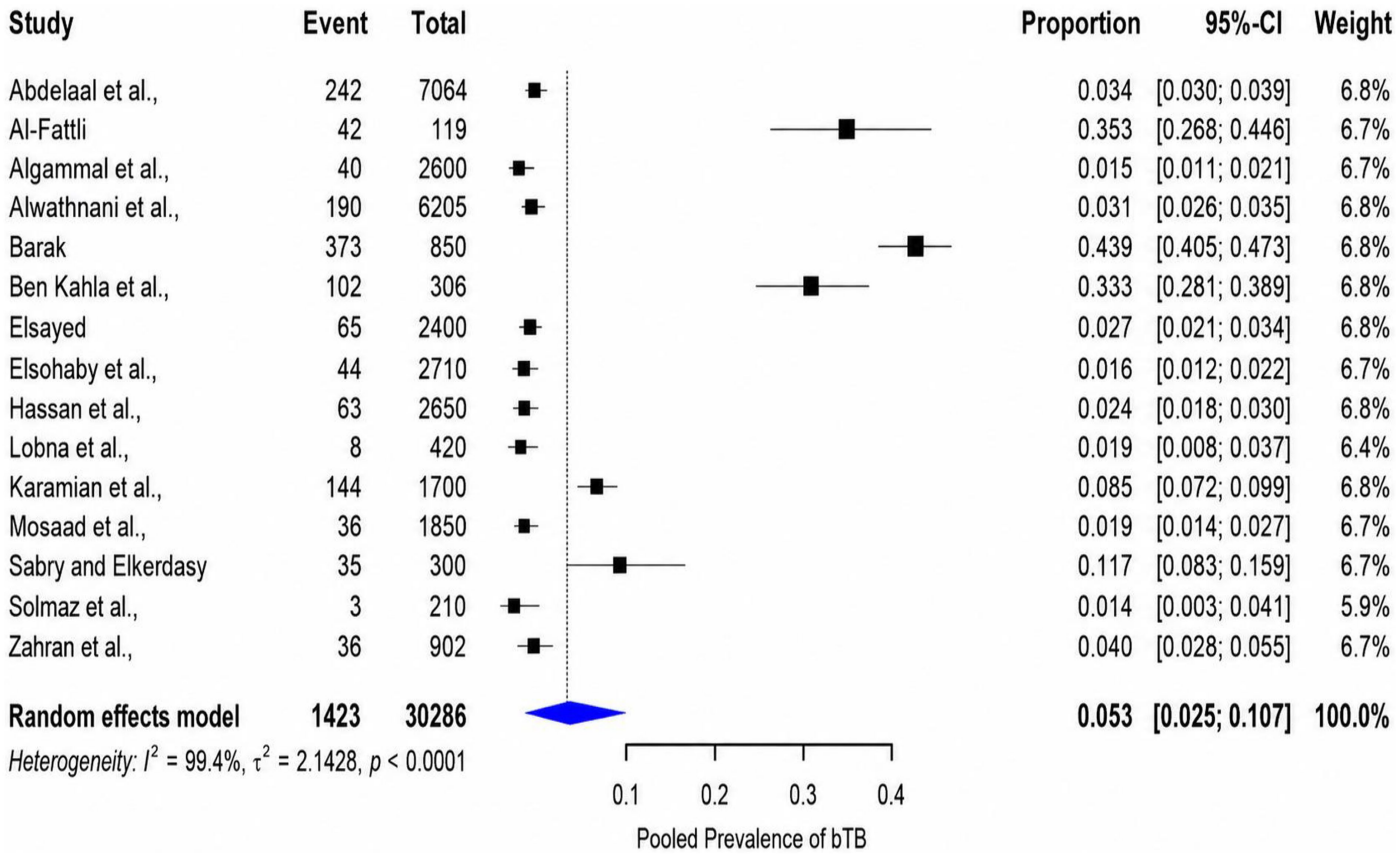
Forest plot of pooled bTB prevalence using the tuberculin skin test (TST) screening method. The forest plot presents the pooled prevalence estimates of bTB among animals screened using the tuberculin skin test (TST). Individual study prevalence estimates ranged from 1.4 to 43.9%. The random-effects model estimated an overall pooled prevalence of 5.3% (95% CI: 2.5–10.7%) based on 15 studies involving 30,286 animals and 1,423 positive cases. Substantial heterogeneity was observed among studies (*I*^2^ = 99.4%, τ^2^ = 2.1428, *p* < 0.001).

Of the 38 studies included in the meta-analysis, 15 studies used TST as a screening method of bTB. These studies involved 30,286 animals, of which 1,423 tested positive for bTB. The pooled prevalence estimated using the random-effects model was 5.3% (95% CI: 2.5–10.7%). Individual study estimates showed substantial variation, ranging from 1.4 to 43.9%, as illustrated in the forest plot (Figure 2). Extremely high heterogeneity was observed among studies (I^2^ = 99.4% and tau^2^ = 2.1428). The heterogeneity test was statistically significant (*p* < 0.001), indicating considerable variability across studies.

#### 3.2.1 Sensitivity analysis of studies done by screening test

**Figure 3:**
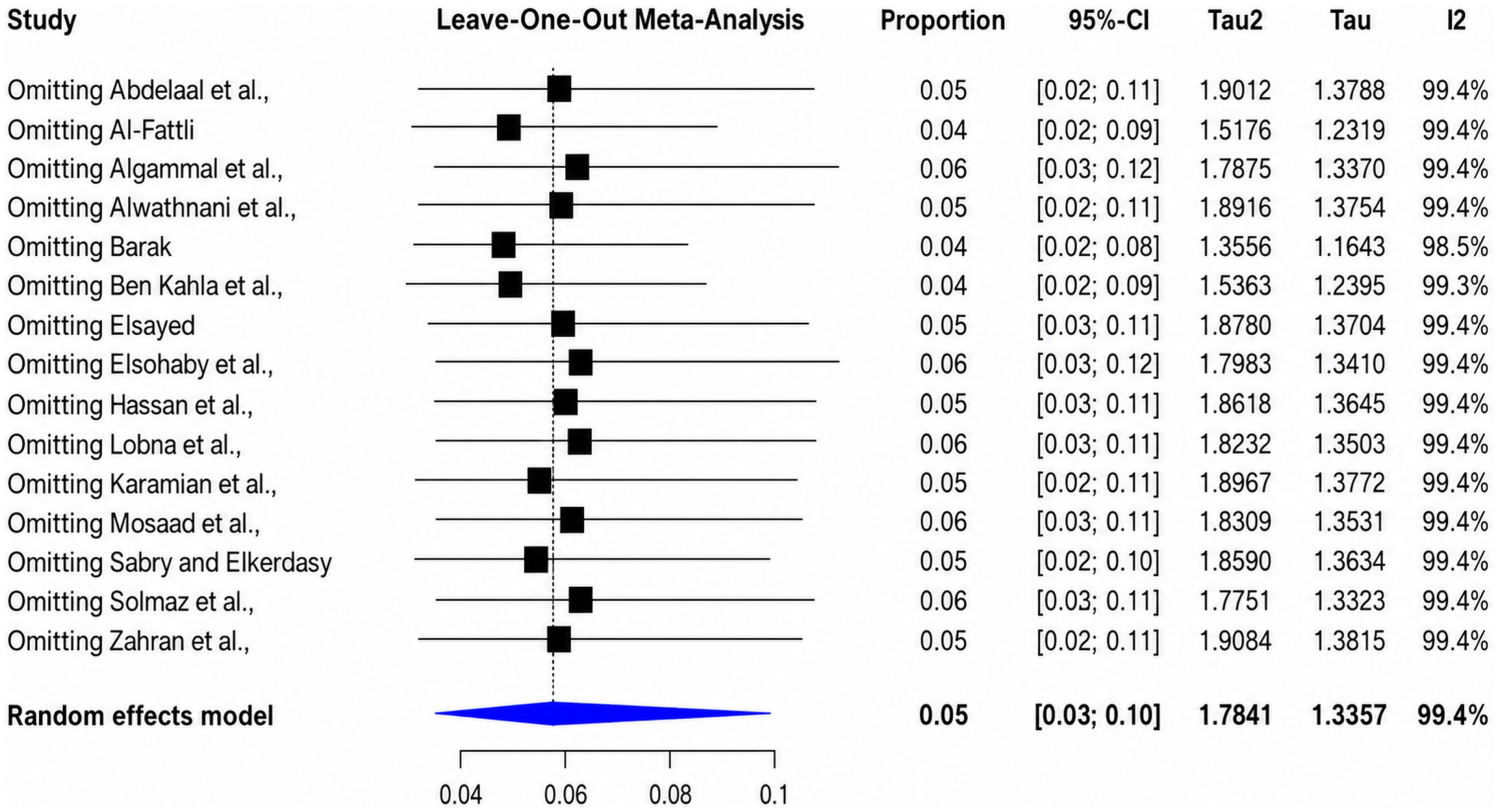
Screening test-based sensitivity analysis forest plot of bTB in MENA region. This figure shows that removing individual studies did not meaningfully change the pooled prevalence estimate (0.04–0.06) or heterogeneity (*I*^2^ = 98.5–99.4%), indicating that the meta-analysis results were stable and robust.

The leave-one-out sensitivity analysis demonstrated that omission of individual studies did not substantially alter the pooled prevalence that was estimated by the screening test (TST) (Figure 3). The pooled prevalence remained relatively stable, ranging from 4 to 6% after sequential exclusion of each study, with the overall random-effects estimate remaining approximately 5% (95% CI: 3–10%). Similarly, heterogeneity remained consistently high across all iterations, with I^2^ values ranging from 98.5 to 99.4%. None of the excluded studies produced a marked reduction in heterogeneity or a significant shift in the pooled estimate, indicating that no single study disproportionately influenced the overall meta-analysis results. These findings suggest that the pooled prevalence estimate was robust and that the observed heterogeneity was likely attributable to genuine differences among studies rather than the effect of any individual study.

#### 3.2.2 Assessment of publication bias of studies done by screening test

**Figure 4:**
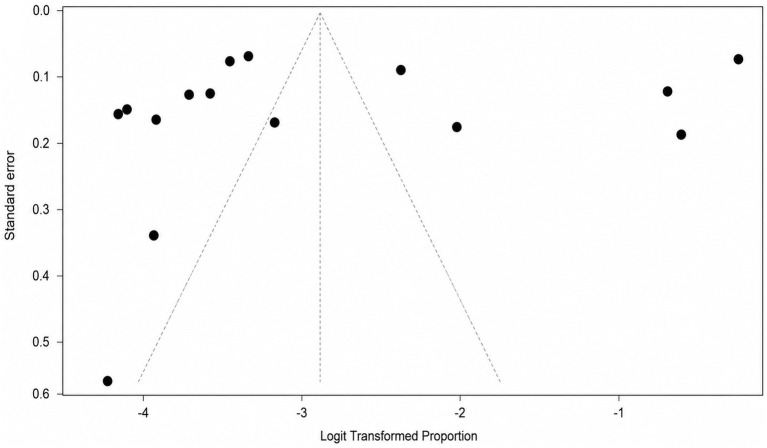
Funnel plot showing publication bias among 15 meta-analysis studies on bTB in animals in the MENA region. The funnel plot shows the relationship between the logit-transformed proportion of bTB prevalence in animals and the standard error across 15 studies conducted in the MENA region. Each black circle represents an individual study, the vertical dotted line indicates the pooled effect estimate, and the diagonal dashed lines represent the 95% confidence limits. Studies with smaller standard errors are located at the top, while those with larger standard errors appear toward the bottom of the plot.

The funnel plot demonstrates an asymmetric distribution of studies around the pooled effect estimate. Several studies are concentrated on the left side of the plot, while comparatively fewer studies appear on the right side. In addition, the number of smaller studies with larger standard errors are unevenly dispersed outside the funnel boundaries. This unequal distribution suggests the possible presence of publication bias or small-study effects among the included studies evaluating animal bTB prevalence in the MENA region (Figure 4).

Egger’s regression test was conducted to evaluate the presence of publication bias and the test revealed no statistically significant evidence of funnel plot asymmetry (z = −1.2773, *p* = 0.2015), suggesting that publication bias was not substantial among the 15 included studies. Furthermore, the estimated effect size as the standard error approached zero was −2.2640 (95% CI: −3.4426 to −1.0854). These findings indicate that the pooled prevalence estimate was relatively stable and not significantly influenced by small-study effects or selective publication.

### 3.3 Pooled prevalence of bovine tuberculosis on the basis of confirmatory test

**Figure 5:**
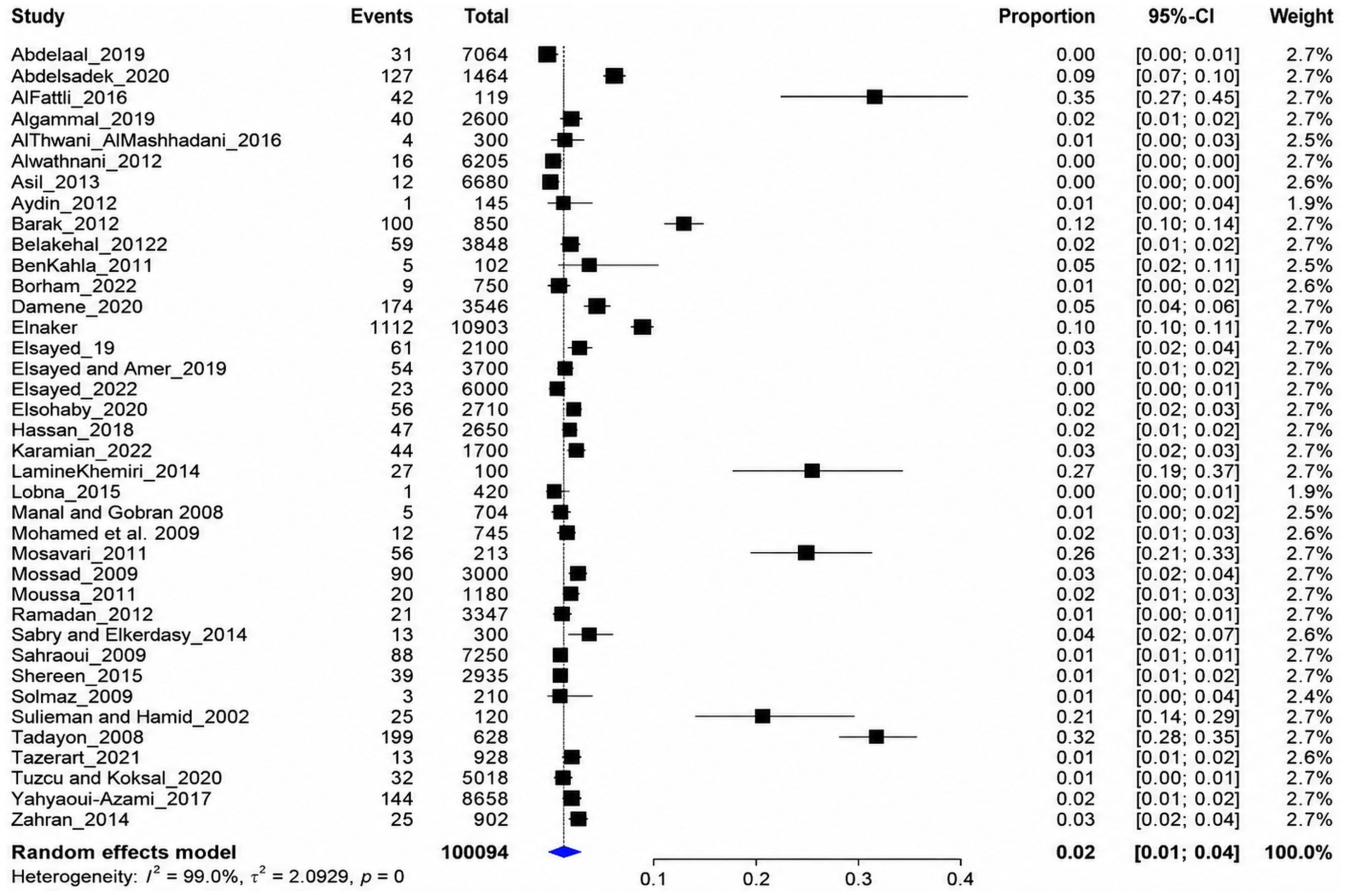
Forest plot of pooled bTB prevalence based on confirmatory diagnostic tests across 38 studies in the MENA region. The forest plot presents the pooled prevalence estimates of bTB based on confirmatory diagnostic tests from 38 studies involving 100,094 animals. Individual study prevalence estimates ranged from less than 1 to 35%. The random-effects model estimated an overall pooled prevalence of 2.3% (95% CI: 1–4%). Considerable heterogeneity was observed among studies (*I*^2^ = 99.0%, τ^2^ = 2.0929, *p* < 0.001).

The meta-analysis included 100,094 animal observations, of which 2,830 were positive for bTB. Using a random-effects logistic regression model, the pooled prevalence of bTB in animals across the MENA region was estimated at 2.3% (95% CI: 1.43–3.69%). A substantial degree of heterogeneity was observed among the included studies. The between-study variance was estimated at *τ*^2^ = 2.0960, with a corresponding τ value of 1.4477. The inconsistency index was extremely high (I^2^ = 99.0%; 95% CI: 98.8–99.1%), indicating considerable variability between studies beyond chance alone. Similarly, the H statistic was 9.84 (95% CI: 9.28–10.44), further confirming marked heterogeneity (Figure 5). The heterogeneity tests demonstrated statistically significant variation among studies, as shown by both the Wald test (*Q* = 3582.88, df = 37, *p* < 0.001) and the likelihood ratio test (LRT = 4246.26, df = 37, *p* < 0.001). These findings suggest notable differences in bTB prevalence estimates across studies conducted in the MENA region, potentially reflecting variations in animal species, diagnostic approaches, geographical locations, and study designs.

#### 3.3.1 Geographic region-based subgroup analysis of pooled prevalence

**Figure 6:**
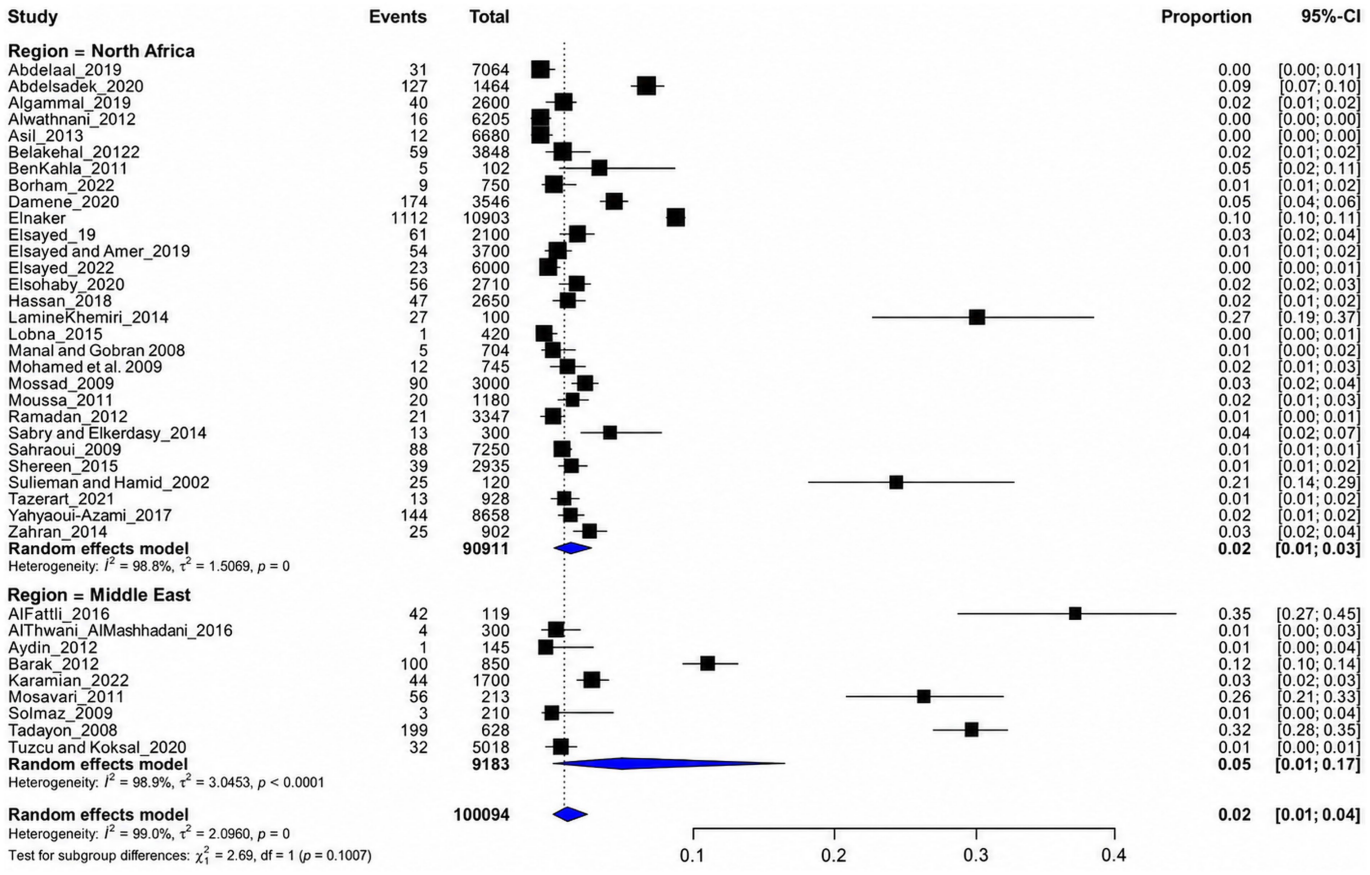
Forest plot of geographic region-based subgroup analysis pooled prevalence of bovine tuberculosis. Subgroup forest plot comparing pooled bTB prevalence between North Africa and the Middle East, showing prevalence estimates of 2% (95% CI: 1–3%) and 5% (95% CI: 1–17%), respectively.

Subgroup analyses were performed according to predefined variables, including geographic region and host species. Geographic region-based subgroup meta-analysis was conducted to evaluate regional differences in the prevalence of bTB among animals across the MENA region (Figure 6). Using the random-effects model, the pooled prevalence of bTB in North Africa was estimated at 2% (95% CI: 1–3%). Although the pooled prevalence was relatively low, substantial heterogeneity was observed among the included studies (*I*^2^ = 98.8%, *p* < 0.001). Most studies conducted in North Africa reported prevalence estimates below 5%; however, a few studies showed markedly higher prevalence values ranging from approximately 21 to 27%, contributing to the high between-study variability.

In the Middle East subgroup, the pooled prevalence of animal bTB was estimated at 5% (95% CI: 1–17%). Considerable heterogeneity was also detected within this subgroup (I^2^ = 98.9%; χ^2^ = 61.54; *p* < 0.0001). The broader confidence interval observed in the Middle East subgroup indicates greater variability among the included studies compared to North Africa. The pooled prevalence estimate in the Middle East was higher than that reported in North Africa, although heterogeneity remained extremely high in both geographic regions.

#### 3.3.2 Egyptian and other MENA countries-based subgroup analysis of pooled prevalence

**Figure 7:**
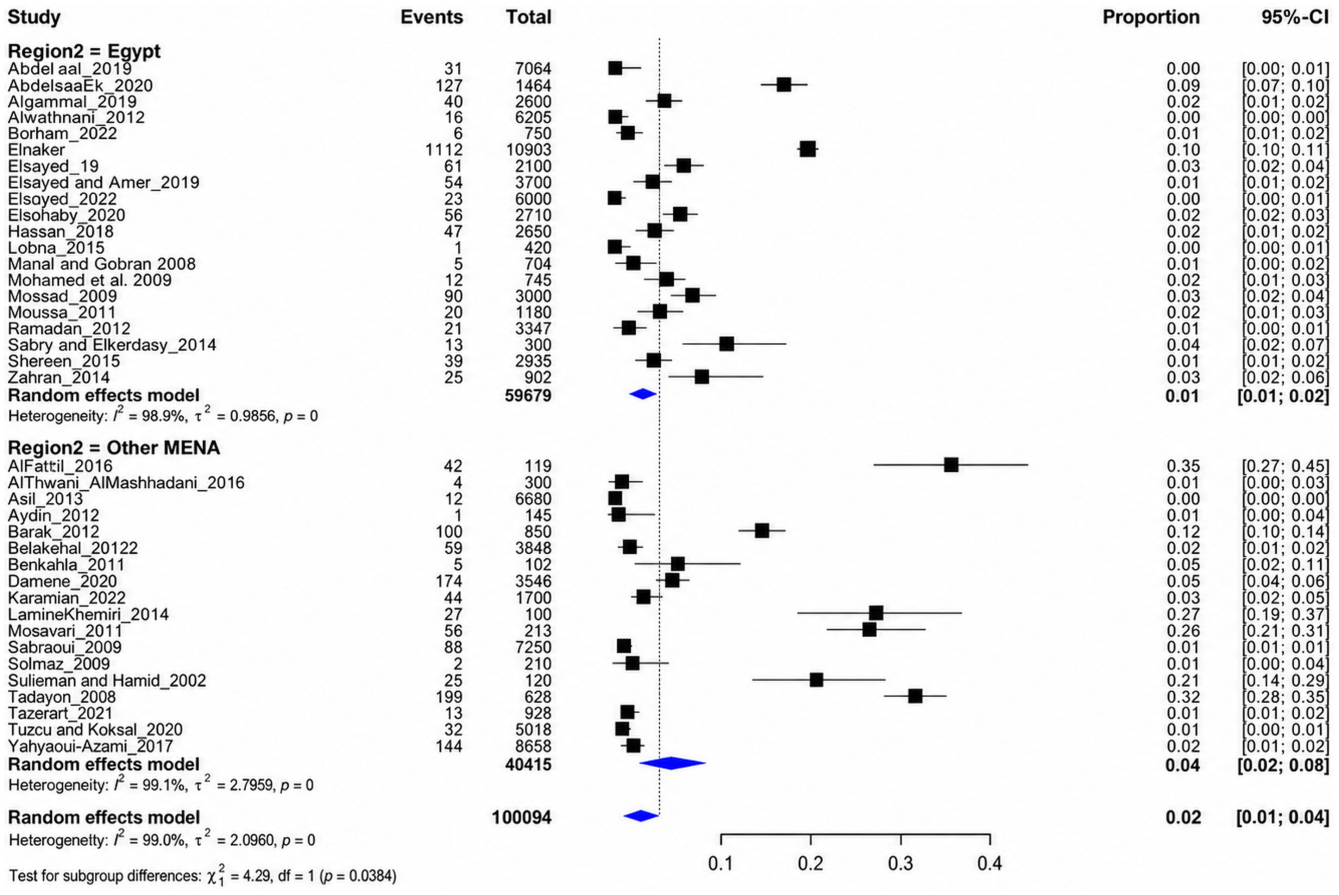
Forest plot of Egyptian and other MENA countries studies-based subgroup analysis of pooled prevalence of bovine tuberculosis. The forest plot shows the pooled prevalence of bTB in animals from 38 studies conducted in the MENA region, grouped into Egypt and other MENA countries. Squares represent individual study estimates, horizontal lines indicate 95% confidence intervals, and diamond shapes show pooled prevalence estimates for each subgroup and the overall analysis using a random-effects model.

Egyptian and other MENA countries-based subgroup analysis showed that studies conducted in Egypt had a pooled prevalence of 1% (95% CI: 1–2%) while the pooled prevalence in other MENA countries was 4% (95% CI: 2–8%). Considerable heterogeneity was observed among the included studies in both subgroups. The heterogeneity values were high in both Egyptian studies (I^2^ = 98.9%) and in studies from the other MENA countries (I^2^ = 99.1%). The difference in pooled prevalence between the Egyptian studies and studies in the other MENA countries was significant (χ^2^ = 4.29, df = 1, *p* = 0.0384) (Figure 7).

#### 3.3.3 Animal species-based subgroup analysis of pooled prevalence

Animal species-based subgroup analysis showed that the pooled prevalence in cattle was 2% (95% CI: 1–4%). Considerable heterogeneity was observed among studies conducted in cattle (*I*^2^ = 98.8%, *p* < 0.001). Although most cattle studies reported prevalence values below 5%, some studies reported markedly higher prevalence estimates reaching 27–35%, contributing to the high heterogeneity.

In the mixed-species subgroup (cattle and buffalo), the pooled prevalence was 2% (95% CI: 0–7%). Similar to the cattle subgroup, heterogeneity was extremely high (I^2^ = 99.4%, *p* < 0.0001). Several mixed-species studies reported relatively high prevalence estimates, reaching 32%, which resulted in wider confidence intervals and increased variability between studies. However, there was no significant difference between studies pooled prevalence in conducted in cattle and studies conducted mixed-species (χ^2^ = 0.24, df = 1, *p* = 0.8252, Forest plot is not shown).

#### 3.3.4 Sensitivity analysis of prevalence values reported by the confirmatory test

**Figure 8:**
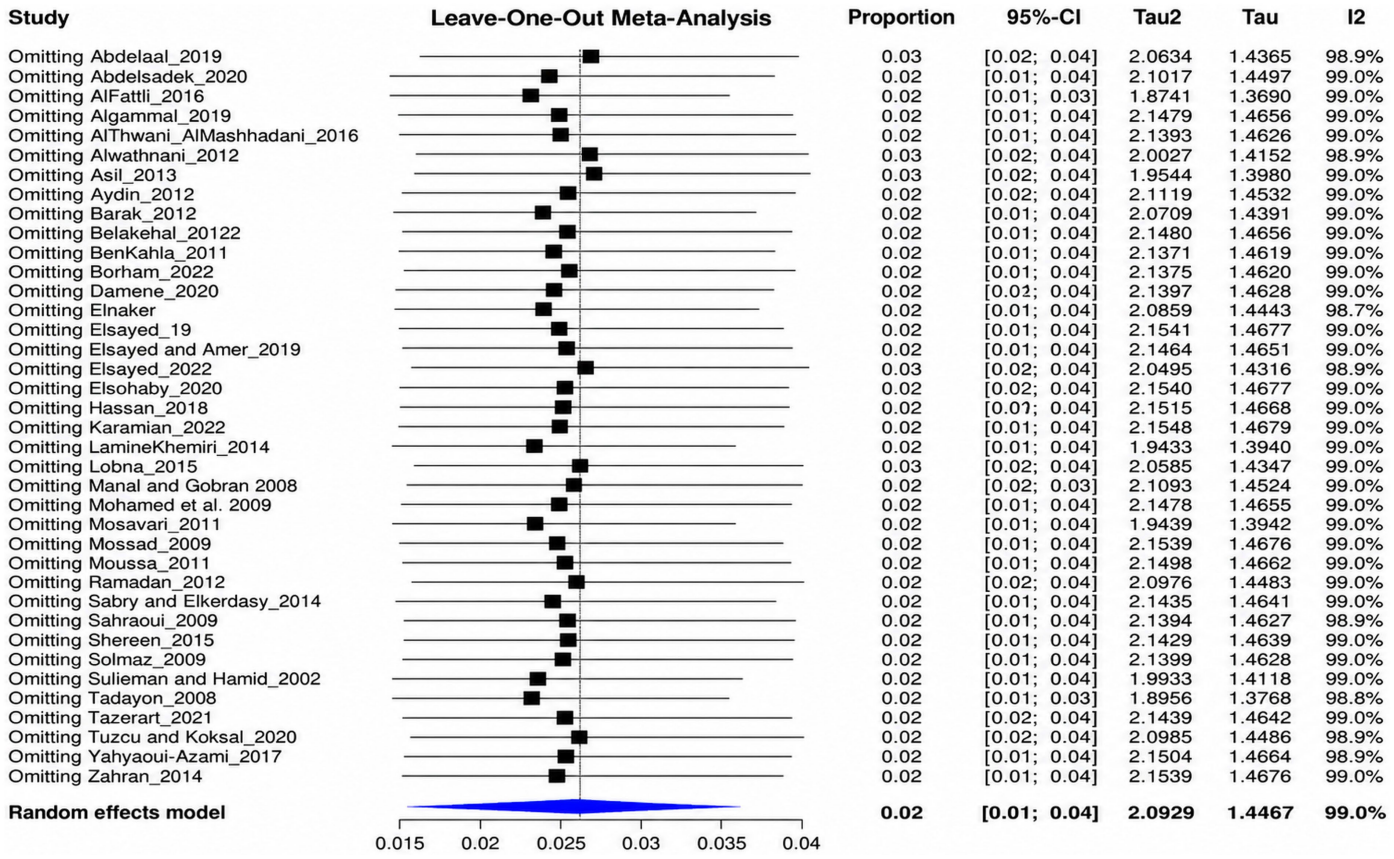
Sensitivity analysis of pooled prevalence of bovine tuberculosis in the MENA region based on confirmatory test. The figure presents a leave-one-out sensitivity analysis assessing the stability of the pooled bTB prevalence estimate across MENA studies. Sequential exclusion of individual studies showed minimal changes in the pooled estimate, indicating robust overall results.

The leave-one-out sensitivity analysis demonstrated that exclusion of any single study did not substantially alter the overall pooled prevalence estimate. The pooled prevalence remained relatively stable at approximately 2% (95% CI: 1–4%) throughout all iterations of the analysis. Similarly, the heterogeneity values remained consistently high across the analysis, with I^2^ values ranging from 98.7 to 99.0%. None of the individual studies exerted a disproportionate influence on the overall meta-analysis result, as the recalculated pooled estimates and confidence intervals showed only minimal variation after sequential omission of each study. These findings indicate that the overall pooled prevalence estimate was robust and not driven by any single study included in the meta-analysis (Figure 8).

#### 3.3.5 Sensitivity analysis of prevalence values reported by confirmatory test excluding Egyptian studies

**Figure 9:**
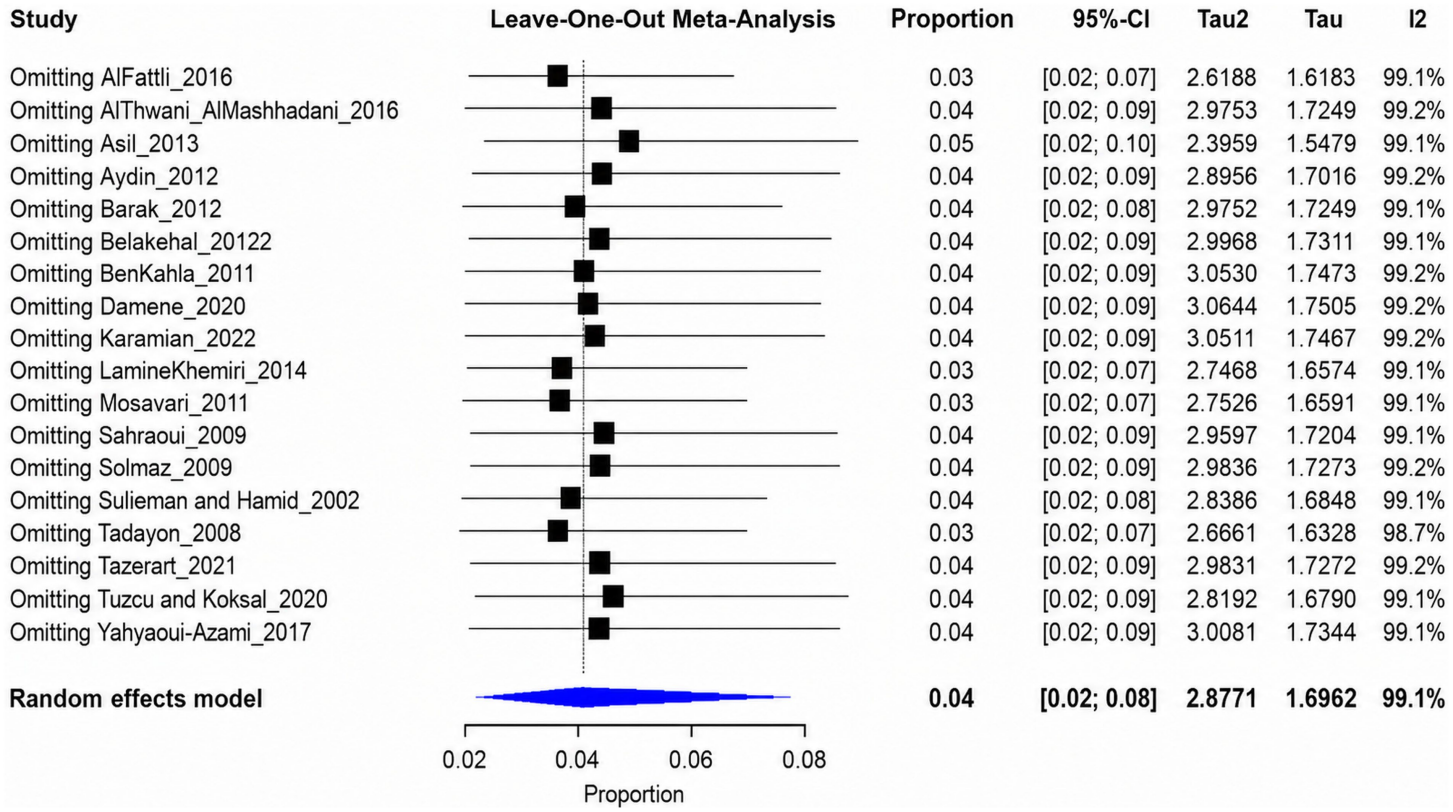
Sensitivity analysis of pooled prevalence of bovine tuberculosis by confirmatory test in the MENA region excluding Egypt. The figure presents a leave-one-out sensitivity analysis performed after excluding studies conducted in Egypt to assess the robustness of the pooled prevalence estimate of bovine tuberculosis (bTB) in animals across the remaining MENA countries. Each row represents the pooled prevalence estimate recalculated after omitting one study at a time. Squares indicate the pooled prevalence estimates, while horizontal lines represent the corresponding 95% confidence intervals (CIs). The blue diamond at the bottom represents the overall pooled estimate generated from the random-effects model.

The sensitivity analysis excluding Egypt-based studies showed that the pooled prevalence estimates remained relatively stable throughout the sequential omission of individual studies. The recalculated pooled prevalence ranged from 3 to 5%, with most estimates centered around 4% (95% CI: 2–8%) (Figure 9). This indicates that no single study substantially influenced the overall pooled prevalence estimate. Heterogeneity remained consistently high across all iterations of the analysis, with I^2^ values ranging from 98.7 to 99.2%. Similarly, the between-study variance (τ^2^) showed only minor fluctuations after omission of individual studies. These findings demonstrate that the pooled prevalence estimate among non-Egyptian MENA studies was robust and not disproportionately affected by any single study included in the meta-analysis.

#### 3.3.6 Meta-regression analysis of prevalence values reported by the confirmatory test

**Figure 10:**
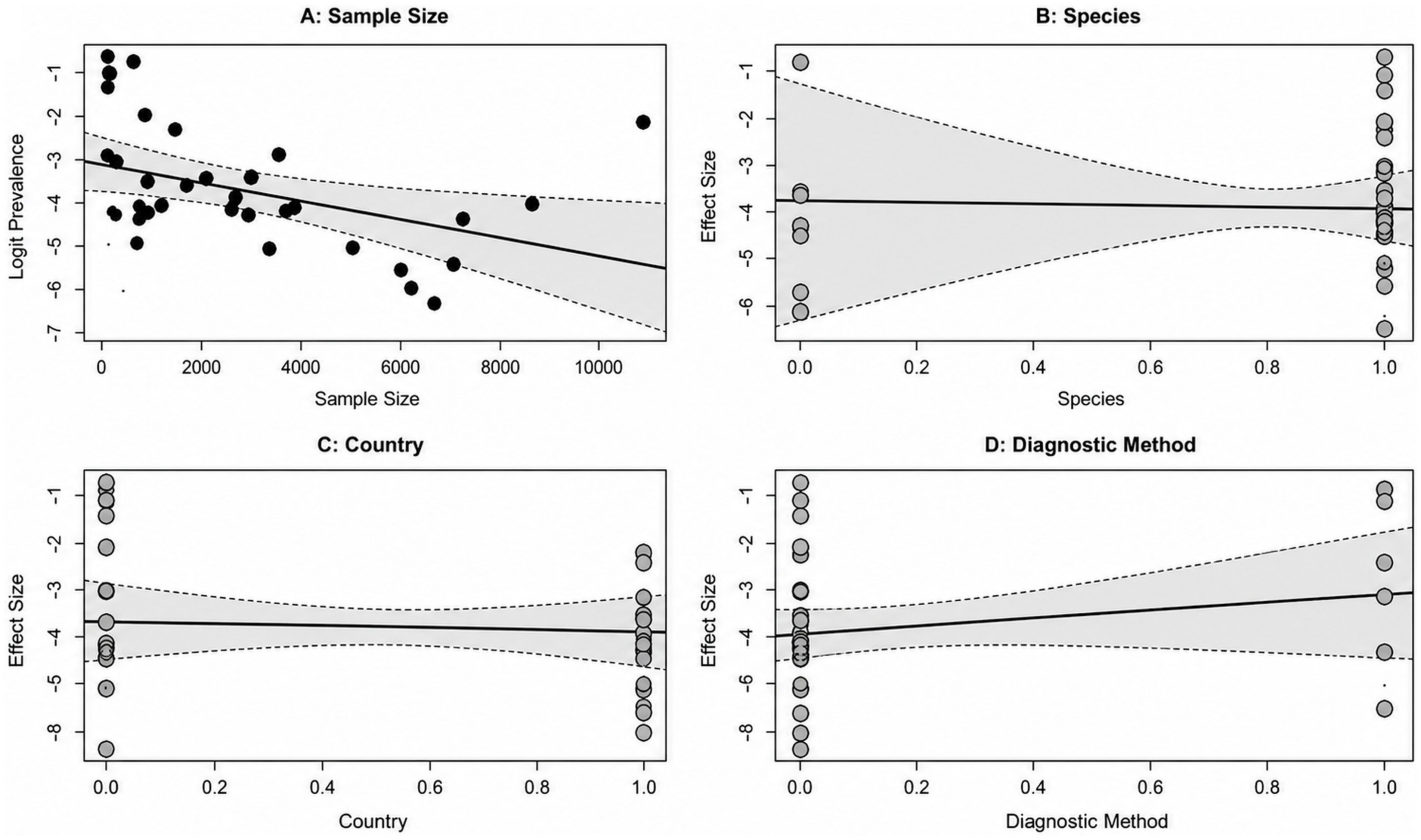
Meta-regression analysis of factors associated with heterogeneity in bTB prevalence across studies in the MENA region. The figure presents mixed-effects meta-regression analyses evaluating the effects of sample size **(A)**, Sample size **(B)** Species **(C)**, Country and **(D)**, Diagnostic methods on bTB prevalence. A significant negative association was observed between sample size and prevalence, while animal species and diagnostic methods showed no significant effects. Geographic variation was significant, with higher prevalence estimates, particularly in Iran. Shaded areas represent the 95% confidence intervals, and substantial residual heterogeneity remained across all models.

Meta-regression was performed using random-effects models with restricted maximum likelihood (REML) estimation to investigate potential sources of heterogeneity in bTB prevalence across the included studies. The evaluated covariates included sample size, animal species, diagnostic method, and country of study (Figure 10).

Sample size showed a statistically significant negative association with bTB prevalence (QM = 6.46, *p* = 0.011). The regression coefficient indicated that studies with larger sample sizes tended to report slightly lower prevalence estimates (*β* = −0.0002; 95% CI: −0.0004 to −0.0000). Sample size explained 14.7% of the between-study heterogeneity; however, substantial residual heterogeneity remained (*I*^2^ = 98.76%), suggesting that additional factors contributed to the observed variability.

Animal species was not significantly associated with prevalence variation (QM = 0.39, *p* = 0.824). Neither cattle-only nor mixed cattle–buffalo studies differed significantly from the reference category, and the model explained none of the observed heterogeneity (R^2^ = 0%). Residual heterogeneity remained extremely high (*I*^2^ = 99.19%).

Diagnostic method was not significantly associated with pooled prevalence estimates (QM = 7.52, *p* = 0.185). None of the diagnostic categories differed significantly from the reference group. Overall, diagnostic method explained only 7.05% of the heterogeneity, while substantial unexplained variability persisted (*I*^2^ = 98.98%).

Country of study was identified as a significant source of heterogeneity (QM = 19.32, *p* = 0.007), accounting for 26.24% of the between-study variability. Compared with the reference category, significantly higher prevalence estimates were observed in Iran (*β* = 2.14, *p* = 0.026), while Iraq and Tunisia showed borderline significance. No statistically significant differences were found for Egypt, Morocco, Sudan, or Turkey. Despite this, residual heterogeneity remained high (*I*^2^ = 98.67%).

In the full multivariable meta-regression model including sample size, species, country, and diagnostic method simultaneously, the combined moderators were not statistically significant overall (QM = 21.89, *p* = 0.111). The model explained 16.57% of the heterogeneity, indicating that the included covariates only partially accounted for the substantial variability observed among studies.

#### 3.3.7 Publication bias assessment prevalence values reported by the confirmatory test

**Figure 11:**
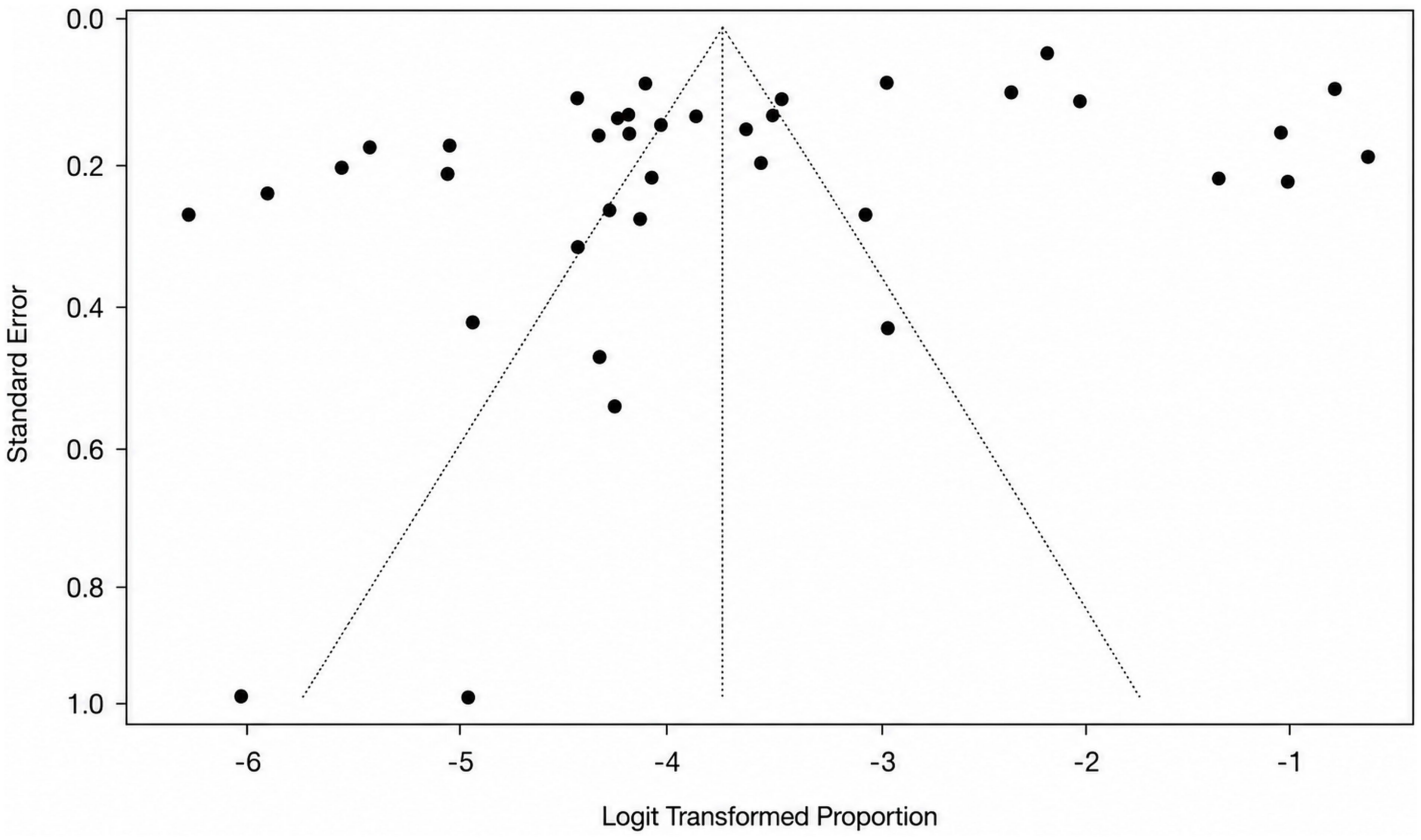
Funnel plot assessing publication bias among 38 studies included in the meta-analysis of bTB prevalence in animals across the MENA region. The figure shows funnel plot asymmetry for bTB prevalence studies, suggesting potential publication bias and small-study effects among the included studies.

Visual inspection of the funnel plot showed a somewhat asymmetric distribution of studies around the pooled prevalence estimate. Several studies were unevenly scattered on both sides of the funnel, with some smaller studies appearing outside the expected funnel boundaries. This pattern suggests the possible presence of publication bias or small-study effects among the included studies. In addition, the wide dispersion of studies reflects substantial heterogeneity in prevalence estimates across the 38 studies included in the meta-analysis (Figure 11).

#### 3.3.8 Egger’s regression test for publication bias of prevalence values reported by confirmatory test

Assessment of small-study effects using Egger’s regression test demonstrated statistically significant funnel plot asymmetry: *t* = −3.50, df = 36, *p* = 0.0012. The negative bias coefficient (−7.87) suggests that smaller studies tended to report higher or more extreme prevalence estimates compared with larger studies. This pattern is consistent with the presence of publication bias and/or small-study effects, indicating that studies with lower or non-significant prevalence estimates may be underrepresented in the literature. However, given the extremely high heterogeneity, part of the observed asymmetry may also be explained by true differences between studies rather than publication bias alone.

#### 3.3.9 Trim-and-fill analysis prevalence values reported by the confirmatory test

To further assess and adjust for potential publication bias, a trim-and-fill procedure was performed. The analysis imputed 14 potentially missing studies, resulting in an adjusted dataset of 52 studies in total. After correction, the pooled prevalence slightly increased to 0.0591 (95% CI: 0.0341–0.1007). This adjusted estimate suggests that the presence of missing studies may have led to an underestimation of the true pooled prevalence in the original analysis. The imputed studies were predominantly positioned on the right side of the funnel plot, indicating that smaller studies with higher prevalence values may be underreported or missing. Despite the adjustment, heterogeneity remained extremely high *I^2^* = 99.1 %. This confirms that substantial variability persists even after accounting for potential publication bias.

### 3.4 *M. bovis* clonal complexes

#### 3.4.1 Characteristics *M. bovis* clonal complexes identified so far

So far, five *M. bovis* clonal complexes have been identified, which include African 1 (Af1), Af2, Eu1, Eu 2 and Eu 3 (Table 2). The representative Spoligotyping patterns of the five clonal complexes are presented in Table 2 to elaborate on the characteristics of the five *M. bovis* clonal complexes. For example, SB0944 represents Af1 clonal complex, which is characterized by the deletion of spacer 30. This clonal complex has been predominantly reported from west African countries (Nigeria, Cameroon, Chad, Mali, and Burkina Faso). While SB0133 belongs to the African 2 (Af2) clonal complex, which is characterized by deletions of spacers 4–7. Af2 strains have been isolated east African countries (Ethiopia, Uganda, Tanzania, and Burundi). Regarding European strains, SB0140 represents the Eu1 clonal complex, which is characterized by the deletion of spacer 11. Eu1 strains showed a broad geographic distribution and have been reported from the United Kingdom, Ireland, the United States, Mexico, and New Zealand. Besides, SB0121 represents the Eu2 clonal complex which is characterized by the deletion of spacer 21. This clonal complex has been reported from southern and western Europe (Spain, Portugal, and France), Middle Eastern countries (Turkey and Iran) and North African countries (Morocco and Algeria). Eu3 was recently shown to be predominant in Western Europe and East-Africa.

**Table 2:** Representative spoligotype patterns, spacer deletions, clonal complexes, and geographic distribution of major *M. bovis* lineages.

**Clonal complex**	**Representative spoligotypes**	**Spacers**	**Deleted Spacers**	**Geographic distribution**	**References**
Af1	SB0944	1101111101111110111111111111101111111100000	30	West Africa	56
Af2	SB0133	1100000101111110111111111111111111111100000	4 to 7	East Africa	57,58
Eu1	SB0140	1101111101011110111111111111111111111100000	11	Global	11
Eu2	SB0121	1101111101111110111101111111111111111100000	21	Europe, Middle East and North Africa	59
Eu3	–	Defined by SNP markers, not by spacer deletions	SNP	Western Europe and East-Africa	60

#### 3.4.2 *M. bovis* clonal complexes reported from the Middle East and North Africa

**Figure 12:**
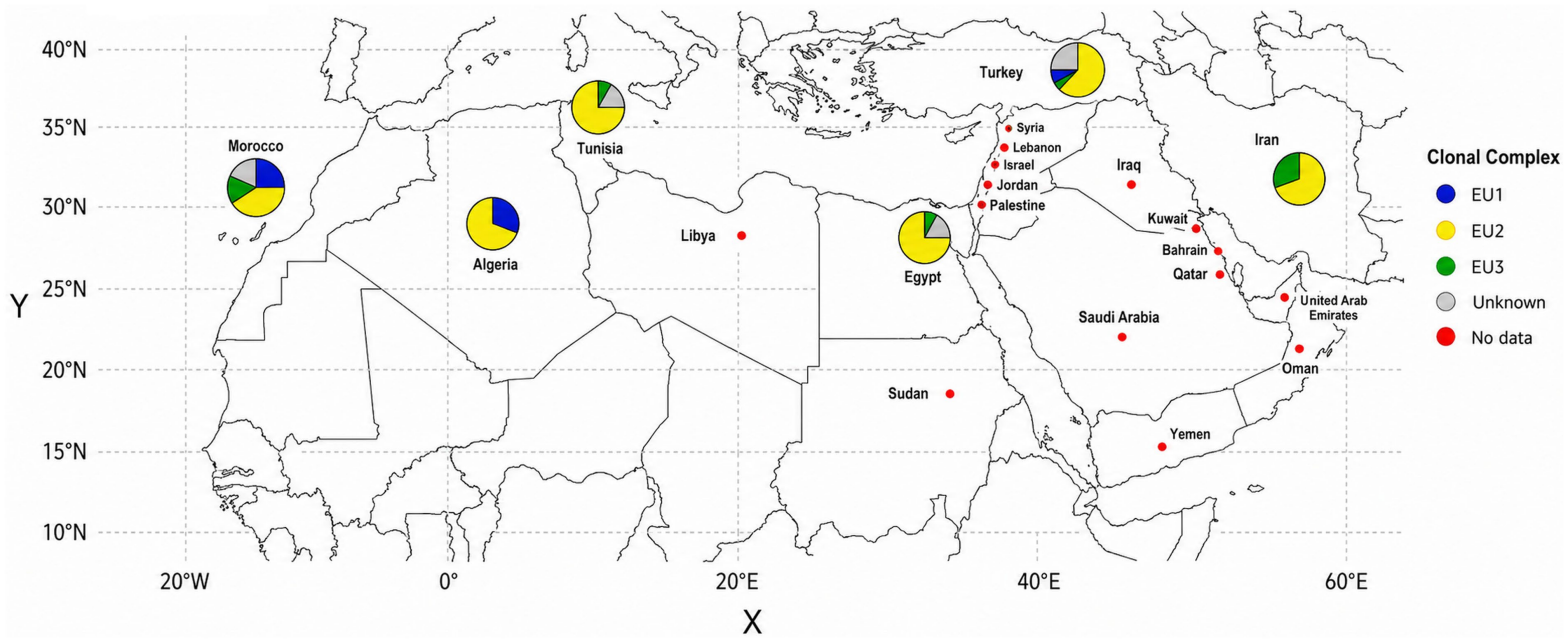
Geographic distribution of *Mycobacterium bovis* clonal complexes across MENA countries. The figure illustrates the distribution of *M. bovis* clonal complexes among MENA countries. Pie charts represent the relative proportions of EU1, EU2, EU3, and unknown clonal complexes reported in each country, while red dots indicate countries with no available molecular data. EU2 was the predominant clonal complex across most countries, whereas EU1 and EU3 showed more limited geographic distribution.

Based on the results of studies reported from the MENA region, 590 *M. bovis* isolates were reported between 2000 and 2025, and 63.6% of the isolates were classified as Eu2 isolates while 9.7 and 6.6% were classified as Eu1 and Eu3, respectively (Figure 12). However, 20.2% of the isolates could not be classified under the presently known *M. bovis* clonal complexes. In terms of spoligotype patterns, SB0120, SB0121, and SB0134 were the most frequently reported spoligotypes from the region (Table 3).

**Table 3:** Studies conducted on the molecular typing of *M. bovis* in the MENA Region (2000–2025).

**Country**	**No. of Isolates**	**Major Spoligotypes**	**Eu1**	**Eu2**	**Eu3**	**Unknown**	**Molecular Methods**	**Reference**
Morocco	134	SB0121, SB0265, SB0120	20 (15%)	60 (45%)	18 (13%)	36 (27%)	Spoligotyping, RD typing	54
Morocco	55	SB0121, SB0265, SB0120	12 (21.8%)	23 (41.8%)	6 (10.9%)	14 (25.5%)	Whole-Genome Sequencing (WGS)	61
Morocco	6	SB0121	0 (0%)	3 (50%)	0 (0%)	3 (50%)	WGS, PCR	62
**Overall**	**195**		**32 (16.4%)**	**86 (44.1%)**	**24 (12.3%)**	**53 (27.2%)**		
Algeria	80	SB0121, SB0120	25 (31%)	55 (69%)	0 (0%)	0 (0%)	Spoligotyping, VNTR	63
Tunisia	43	SB0120, SB0134	0 (0%)	31 (72%)	0 (0%)	12 (28%)	Spoligotyping	15
Iran	132	SB0120, SB0121	0 (0%)	98 (74%)	0 (0%)	34 (26%)	Spoligotyping, MIRU-VNTR	64
Turkey	100	SB0120, SB0140	0 (0%)	65 (65%)	15 (15%)	20 (20%)	Spoligotyping, MIRU-VNTR	65
Egypt	40	SB0268	0 (0%)	40 (100%)	0 (0%)	0 (0%)	Whole-Genome Sequencing (WGS)	32
**Overall**	**590**		**57 (9.7%)**	**375 (63.6%)**	**39 (6.6%)**	**119 (20.2%)**		

Individual country-based data indicated that 195 *M. bovis* isolates were reported from Morocco, most of which (44.1%) were classified in Eu2 clonal complex while Eu1 and Eu3 shared 16.4 and 12.3% and the remaining 27.2% were unclassified. In Algeria, 69% of 80 isolates were grouped under Eu2 while the remaining 31% were Eu1. In Tunisia, Eu2 consisted of 72% the 43 isolates while the remaining 28% could not be classified under the known clonal complexes. In Iran, 74% of the 132 isolates were grouped under Eu2 while the remaining 26% could not be classified under the existing clonal complexes. In Turkey, 65% of the 100 isolates grouped under Eu2 while 15% classified as Eu3 while 20% could not be classified under the existing clonal complexes. In Egypt, all the 40 isolates (100%) were classified as Eu2 clonal complex.

## 4 Discussion

This systematic review and meta-analysis evaluated the prevalence of bTB and the distribution of *M. bovis* clonal complexes across the MENA region. The review included studies that used TST, culture, PCR, and molecular typing methods. The pooled prevalence was 5.3% (95% CI: 2.5–10.7%) on the basis of screening test and 2.3% (95% CI: 1.43–3.69%) on the basis of confirmatory test. Considerable heterogeneity was observed across studies (I^2^ > 99%) based on confirmatory test. Subgroup analyses revealed regional variation in bTB prevalence between North African and Middle Eastern countries, as well as between Egyptian studies and studies in the other MENA countries, where the pooled prevalence in Egyptian studies was significantly lower prevalence than the pooled prevalence recorded in the other MENA countries. Confirmatory test- based sensitivity analyses confirmed the robustness of the pooled prevalence estimates, whereas funnel plot asymmetry and Egger’s regression test suggested possible publication bias and small-study effects. Meta-regression identified country’s prevalence and sample size as major contributors to heterogeneity. Molecular findings demonstrated predominance of the European 2 (Eu2) clonal complex, while SB0120, SB0121, and SB0134 were the most frequently reported spoligotypes.

The higher pooled prevalence recorded by TST screening test as compared to the pooled prevalence recorded by confirmatory. Similar discrepancies have been reported by systematic reviews from Africa and Asia, where TST-based estimates were frequently higher because of cross-reactivity with environmental mycobacteria and prior sensitization (8, 11, 18). On the other hand, confirmatory techniques such as culture and PCR may underestimate the true burden because of reduced sensitivity in animals with low bacterial load, latent infection, or intermittent shedding. Limited surveillance systems, underreporting, and inconsistent laboratory capacity across several MENA countries may have further contributed to underestimation of the regional disease burden.

In North America, national control programs have reduced bTB prevalence to very low levels in commercial cattle populations in the United States and Canada; however, sporadic outbreaks linked to wildlife reservoirs, particularly white-tailed deer and elk, continue to threaten eradication efforts (19). Similar epidemiological patterns have been observed in New Zealand, where wildlife reservoirs such as possums have played a major role in sustaining transmission despite aggressive control campaigns (5). Compared to the burden of bTB in developed western countries, many MENA countries still lack comprehensive surveillance systems, coordinated eradication programs, and wildlife monitoring strategies, which may contribute to continued disease circulation.

The pooled prevalence estimate recorded in the present review was also lower than reports from parts of Latin America and South America. Studies from Brazil, Argentina, and Mexico have reported herd-level prevalence ranging from 5% to more than 15%, particularly in intensive dairy production systems (13). Similar to the MENA region, heterogeneity in South American studies has been linked to differences in surveillance systems, diagnostic methods, and livestock production practices. In several South American countries, inadequate compensation policies and weak veterinary infrastructure have been identified as major barriers to successful bTB control (20). These challenges resemble those observed in parts of the MENA region, where disease control programs remain fragmented or inconsistently implemented.

Environmental and ecological factors may also contribute to the comparatively lower prevalence observed in some MENA countries. The survival of *M. bovis* is influenced by temperature, humidity, ultraviolet radiation, and environmental contamination. Hot and arid climates characteristic of many MENA countries may reduce bacterial survival in soil, pasture, and water sources, thereby limiting indirect transmission (5). However, climate-related stressors such as drought and heat stress may also weaken host immunity and increase susceptibility to infection under certain production conditions (21).

The pooled prevalence was lower in Egypt than the pooled prevalence recorded in the other MENA countries which could be due to the inclusion of large-scale surveillance studies based on the routine meat inspection procedure which does not consider detailed post mortem examination of all lymph nodes and visceral organs. Moreover, most of the Egyptian studies were conducted on large sample sizes which involved thousands of animals. Meta-regression analysis confirmed a statistically significant negative association between sample size and prevalence estimates, indicating that smaller studies were more likely to report higher prevalence values. Similar small-study effects have been documented in previous infectious disease meta-analyses and may partly reflect publication bias, selective sampling, or localized outbreaks (22).

The other finding of this review was the high heterogeneity across studies (I^2^ > 98%). Similar heterogeneity has been reported in meta-analyses conducted in Africa, Asia, and Latin America, where prevalence estimates vary substantially according to diagnostic methods, study populations, ecological conditions, and surveillance intensity (18, 23). In the present review, country of study and sample size were identified as significant contributors to heterogeneity, whereas animal species and diagnostic method were not significant causes of heterogeneity in the meta-regression analysis model. Nevertheless, most heterogeneity remained unexplained, suggesting the influence of additional epidemiological factors such as herd management, animal movement, wildlife reservoirs, climatic conditions, and differences in veterinary infrastructure.

Subgroup analysis demonstrated significant variation between North African and Middle Eastern countries, with the Middle East showing higher pooled prevalence estimates than the pooled prevalence recorded in North Africa. This variation could reflect differences in cattle production systems, veterinary services, biosecurity implementation, and transboundary livestock movement. Intensive farming systems with high animal density can facilitate rapid disease transmission, whereas extensive production systems may allow spread through communal grazing and unrestricted movement of infected animals. Association between husbandry systems and bTB prevalence has been reported in African and Asian studies (23, 24). Furthermore, cross-border livestock trade remains extensive in several MENA countries, and seasonal animal movement, which may facilitate dissemination of infected animals across national boundaries.

The molecular epidemiological findings demonstrated predominance of European clonal complexes, particularly Eu2 across the MENA region. More than 60% of the typed isolates belonged to Eu2 while Eu1 and Eu3 were less common and African clonal complexes (Af1 and Af2) were absent. Similar dominance of Eu2 has been reported in Southern Europe, particularly Spain, Portugal, and France, where these lineages are historically associated with cattle trade and livestock movement (25). Studies from Morocco, Algeria, Tunisia, Iran, Turkey, and Egypt consistently identified SB0120 and SB0121 as dominant spoligotypes, supporting epidemiological links between Europe and the MENA region. The predominance of European clonal complexes in the MENA region likely reflects historical cattle importation, colonial trade networks, and ongoing livestock exchange with Europe. This observation is consistent with previous phylogeographic studies showing that *M. bovis* lineages are strongly associated with historical animal movement patterns (12). The absence of Af1 and Af2 clonal complexes, which are largely confined to west and east Africa, respectively, may partly be explained by ecological and geographic barriers such as the Sahara Desert that limit northward livestock movement (26). Similar conclusions were highlighted in the review by other authors (24), which emphasized that zoonotic tuberculosis in the MENA region remains neglected despite increasing evidence of transboundary transmission and regional circulation of *M. bovis*. Their review also highlighted weaknesses in integrated One Health surveillance systems, insufficient collaboration between veterinary and human health sectors, and the under-recognition of zoonotic tuberculosis in many MENA countries.

Another important observation is that 20% of isolates could not be classified into currently recognized clonal complexes. Similar observations have recently been reported in genomic studies from Africa and Asia, where whole-genome sequencing identified previously unrecognized *M. bovis* lineages beyond the classical clonal complex framework (25, 27). This finding suggests the possible circulation of regionally adapted or previously undescribed strains in the MENA region and highlights the need for expanded genomic surveillance using high-resolution molecular techniques such as whole-genome sequencing.

The public health implications of these findings are considerable. Although bTB caused by *M. bovis* remains underrecognized in the MENA region, factors such as close human–animal interaction, consumption of unpasteurized milk, informal slaughtering practices, and inadequate food safety measures may increase the risk of bTB transmission. Kasir et al. (27) highlighted zoonotic tuberculosis as a neglected public health issue in the MENA region, largely due to insufficient surveillance systems, limited laboratory capacity, and low awareness among both veterinary and healthcare professionals. Similar observations have been reported in Africa and South Asia, where *M. bovis* contributes substantially to extrapulmonary tuberculosis among high-risk populations (28). These findings underscore the need to strengthen integrated One Health surveillance, expand diagnostic capacity, and improve collaboration between veterinary and public health sectors to reduce the burden of bTB in the region.

## 5 Limitation

This review has several limitations that should be considered when interpreting the findings. Significant heterogeneity was identified among the included studies due to differences in study design, sampled populations, and diagnostic methods, which reduced direct comparability across reports. Variability in the sensitivity and specificity of diagnostic techniques may also have influenced the reported prevalence estimates of bTB.

Language restriction may have introduced publication bias, as the review primarily included studies published in English and may have excluded relevant evidence reported in French, Arabic, or Persian. In addition, the available data were unevenly distributed across the MENA region, with several countries lacking eligible published studies, thereby limiting the regional representativeness of the findings.

Furthermore, differences in surveillance systems, laboratory capacity, and reporting practices among countries reduced the consistency of the epidemiological evidence included in this review. The long study period and the relatively small number of studies within some subgroup categories may also have affected the stability and reliability of certain pooled estimates and subgroup analyses.

## 6 Conclusion

Bovine tuberculosis remains endemic in the MENA region, with a relatively low but variable prevalence across countries and animal populations. The dominance of European clonal complexes, particularly Eu2, highlights the influence of historical livestock movement on disease distribution. Strengthening surveillance systems, standardizing diagnostic approaches, and implementing targeted control strategies are critical to reducing the burden of bTB and minimizing its zoonotic impact.

## Data Availability

The original contributions presented in the study are included in the article/Supplementary material, further inquiries can be directed to the corresponding author/s.
