# Supplementary material for: Bovine tuberculosis in the Middle East and North Africa: a systematic review and meta-analysis on prevalence and Mycobacterium bovis clonal complexes
Source: Front Vet Sci. 2026 Jun 22;13:1861602. doi: 10.3389/fvets.2026.1861602 (PMC13333423; doi:10.3389/fvets.2026.1861602)
Supplement: Supplementary file 1 [file Table_1.docx]

**Supplementary Table S1: Newcastle–Ottawa Scale Quality Assessment**

| **Study No.** | **Author(s)** | **Year** | **Selection (4)** | **Comparability (2)** | **Outcome (3)** | **Total (9)** | **Quality** |
| --- | --- | --- | --- | --- | --- | --- | --- |
| 1 | Sahraoui et al. | 2009 | 3 | 1 | 2 | 6 | Moderate |
| 2 | Belakehal et al. | 2022 | 3 | 2 | 2 | 7 | High |
| 3 | Tazerart et al. | 2021 | 3 | 2 | 3 | 8 | High |
| 4 | Damene et al. | 2020 | 3 | 2 | 3 | 8 | High |
| 5 | Zahran et al. | 2014 | 3 | 1 | 2 | 6 | Moderate |
| 6 | Alwathnani et al. | 2012 | 2 | 1 | 2 | 5 | Moderate |
| 7 | Moussa et al. | 2011 | 3 | 1 | 2 | 6 | Moderate |
| 8 | Ramadan et al. | 2012 | 3 | 1 | 2 | 6 | Moderate |
| 9 | Shereen et al. | 2015 | 2 | 1 | 2 | 5 | Moderate |
| 10 | Manal & Gobran | 2008 | 2 | 1 | 2 | 5 | Moderate |
| 11 | Zahran et al. | 2014 | 3 | 1 | 2 | 6 | Moderate |
| 12 | Lobna et al. | 2015 | 3 | 2 | 3 | 8 | High |
| 13 | Abdellrazeq et al. | 2016 | 3 | 2 | 3 | 8 | High |
| 14 | Elsayed et al. | 2022 | 3 | 2 | 3 | 8 | High |
| 15 | Hassan et al. | 2018 | 3 | 1 | 2 | 6 | Moderate |
| 16 | Borham et al. | 2022 | 3 | 1 | 2 | 6 | Moderate |
| 17 | Elsayed & Amer | 2019 | 3 | 2 | 3 | 8 | High |
| 18 | Abdelsadek et al. | 2020 | 3 | 1 | 2 | 6 | Moderate |
| 19 | Elsayed | 2019 | 3 | 2 | 3 | 8 | High |
| 20 | Mohamed et al. | 2009 | 3 | 1 | 2 | 6 | Moderate |
| 21 | Algammal et al. | 2019 | 3 | 2 | 3 | 8 | High |
| 22 | Mossad et al. | 2009 | 3 | 1 | 2 | 6 | Moderate |
| 23 | Elsohaby et al. | 2020 | 4 | 2 | 3 | 9 | High |
| 24 | Mosavari et al. | 2011 | 3 | 2 | 3 | 8 | High |
| 25 | Mosavari et al. | 2011 | 3 | 2 | 3 | 8 | High |
| 26 | Tadayon et al. | 2006, 2008 | 3 | 2 | 3 | 8 | High |
| 27 | Karamian et al. | 2022 | 3 | 2 | 3 | 8 | High |
| 28 | Al-Thwani & Al-Mashhadani | 2016 | 2 | 1 | 2 | 5 | Moderate |
| 29 | Barak | 2012 | 2 | 1 | 2 | 5 | Moderate |
| 30 | Yahyaoui-Azami et al. | 2017 | 4 | 2 | 3 | 9 | High |
| 31 | Yahyaoui et al. | 2025 | 4 | 2 | 3 | 9 | High |
| 32 | Asil et al. | 2014 | 3 | 1 | 2 | 6 | Moderate |
| 33 | Sulieman & Hamid | 2012 | 2 | 1 | 2 | 5 | Moderate |
| 34 | Aydın et al. | 2016 | 3 | 2 | 3 | 8 | High |
| 35 | Tuzcu & Köksal | 2012 | 3 | 2 | 3 | 8 | High |
| 36 | Solmaz et al. | 2009 | 3 | 2 | 3 | 8 | High |
| 37 | Ben Kahla et al. | 2012 | 3 | 2 | 3 | 8 | High |
| 38 | Lamine-Khemiri et al. | 2014 | 3 | 2 | 3 | 8 | High |

Summary

- High quality (7–9): 21 studies (55.3%)
- Moderate quality (5–6): 17 studies (44.7%)
- Low quality: 0 studies
